# Synergistic Enhancement of Targeted Wound Healing by Near-Infrared Photodynamic Therapy and Silver Metal–Organic Frameworks Combined with S- or N-Doped Carbon Dots

**DOI:** 10.3390/pharmaceutics16050671

**Published:** 2024-05-16

**Authors:** Maja D. Nešić, Iva A. Popović, Jelena Žakula, Lela Korićanac, Jelena Filipović Tričković, Ana Valenta Šobot, Maria Victoria Jiménez, Manuel Algarra, Tanja Dučić, Milutin Stepić

**Affiliations:** 1 Center for Light-Based Research and Technologies COHERENCE, Department of Atomic Physics, Vinča Institute of Nuclear Sciences, National Institute of the Republic of Serbia, University of Belgrade, 11000 Belgrade, Serbia; ivavukicevic@vin.bg.ac.rs; 2 Department of Molecular Biology and Endocrinology, Vinča Institute of Nuclear Sciences, National Institute of the Republic of Serbia, University of Belgrade, 11000 Belgrade, Serbia; pozegaj@vin.bg.ac.rs (J.Ž.); lela@vin.bg.ac.rs (L.K.); 3 Department of Physical Chemistry, Vinča Institute of Nuclear Sciences, National Institute of the Republic of Serbia, University of Belgrade, 11000 Belgrade, Serbia; filipovicj@vin.bg.ac.rs (J.F.T.); ana_v.s@vin.bg.ac.rs (A.V.Š.); 4 Maria Inmaculada School, 29200 Antequera, Spain; mvjimenezherrera@colegiodemariainmaculada.org; 5 Department of Science, INAMAT^2^—Institute for Advanced Materials and Mathematics, Public University of Navarra, 31006 Pamplona, Spain; manuel.algarra@unavarra.es; 6 MIRAS Beamline, ALBA-CELLS Synchrotron, 08290 Cerdanyola del Vallès, Spain; tducic@cells.es

**Keywords:** wound healing, photodynamic therapy, metal–organic frameworks, carbon dots, fibroblasts

## Abstract

The literature data emphasize that nanoparticles might improve the beneficial effects of near-infrared light (NIR) on wound healing. This study investigates the mechanisms of the synergistic wound healing potential of NIR light and silver metal–organic frameworks combined with nitrogen- and sulfur-doped carbon dots (AgMOFsN-CDs and AgMOFsS-CDs, respectively), which was conducted by testing the fibroblasts viability, scratch assays, biochemical analysis, and synchrotron-based Fourier transform infrared (SR-FTIR) cell spectroscopy and imaging. Our findings reveal that the combined treatment of AgMOFsN-CDs and NIR light significantly increases cell viability to nearly 150% and promotes cell proliferation, with reduced interleukin-1 levels, suggesting an anti-inflammatory response. SR-FTIR spectroscopy shows this combined treatment results in unique protein alterations, including increased α-helix structures and reduced cross-β. Additionally, protein synthesis was enhanced upon the combined treatment. The likely mechanism behind the observed changes is the charge-specific interaction of N-CDs from the AgMOFsN-CDs with proteins, enhanced by NIR light due to the nanocomposite’s optical characteristics. Remarkably, the complete wound closure in the in vitro scratch assay was achieved exclusively with the combined NIR and AgMOFsN-CDs treatment, demonstrating the promising application of combined AgMOFsN-CDs with NIR light photodynamic therapy in regenerative nanomedicine and tissue engineering.

## 1. Introduction

The global prevalence of chronic wounds is estimated at 1.51 to 2.21 per 1000 population [1] and has significant implications for healthcare expenditures. In Europe, 4 million patients experience chronic wounds annually [2], and the prevalence rate of chronic non-healing wounds is estimated to be similar to the prevalence rate of heart conditions in developed countries [3]. Whatever the cause, chronic wounds have a significant physical, mental, social, and economic impact on patients and the healthcare system. Up to now, only several chronic wound treatment modalities have been approved by the United States Food and Drug Administration [4,5]. However, a considerable number of patients (44–70%) treated with these therapies still experience non-healing wounds [5]. Consequently, wound healing remains one of the most critical challenges in the medical field, and there is immense pressure on researchers and pharmaceutical companies to develop cost-effective solutions for wound treatment.

Nanoparticles (NPs) have been studied extensively for their potential in wound healing applications [5]. Among the various nanomaterials, silver NPs (AgNPs) are the most effective and widely recognized broad-spectrum antimicrobial agents [6,7]. AgNPs are used in clinical practice for various treatments, such as burns, chronic ulcers, and diabetic wounds that have developed antibiotic resistance and hospital-acquired bacterial infections. Their antimicrobial effect is primarily attributed to releasing silver ions (Ag^+^) after contact with bacteria. The interaction of Ag^+^ with bacterial cell membranes and enzymes disrupts their structure and function, inhibiting and destroying the microorganisms [8]. In addition, it is shown that AgNPs possess immunomodulatory and pro-inflammatory effects and could enhance the healing of surgical wounds by promoting collagen synthesis and angiogenesis [9]. As a result, AgNPs have shown promise as a valuable tool in promoting wound healing and tissue remodeling, making them potential candidates for advanced wound dressing materials and tissue engineering applications. Despite the mentioned beneficial properties of AgNPs, there is a growing interest in enhancing these properties through combination with other substances. One such approach is the integration of Ag^+^ ions with other materials to not only augment their antibacterial effects but also to address common issues like the aggregation of AgNPs and, thus, loss of the active surface area, which frequently occurs upon interaction with bacterial cells [10].

Alongside AgNPs, carbon dots (CDs) hold significant potential as an imaging tool and functional wound dressing for wound clinical management due to their biocompatibility, water solubility, and optical properties (high fluorescence stability, broad excitation spectrum, long adjustable emission wavelength). The recent literature highlights that CDs can reduce wound inflammation, stimulate fibroblast activity, and improve the overall healing environment, offering a promising avenue for enhancing tissue regeneration and repair [11,12,13,14,15,16]. Additionally, the antibacterial properties of CDs, both with and without light enhancement, have already been addressed [17,18,19].

Moreover, metal–organic frameworks (MOFs), the hybrid compound which consists of organic ligands and metal ions, have emerged as a novel component in biomaterial formulations, demonstrating potential in tissue engineering and regenerative medicine [20,21]. MOFs, with their crystalline porous structure, tunable size, good mechanical and chemical stability, and large surface area, offer modular performance for wound healing and skin regeneration by adjusting synthesis steps and conditions, enabling the release of metal ions to influence cellular response and eliminate microorganisms, as well as serving as carriers for bioactive agents. This versatility has led to a surge in research focusing on applying various MOFs to promote wound healing and skin regeneration [22,23,24,25].

NPs can also be used in combination with other therapies for wound healing, such as phototherapy or electrical stimulation. There has been growing interest in using NPs and infrared (IR) light for wound healing in recent years. Specifically, IR light in the near-infrared (NIR) range (780–1300 nm) showed mutual benefits such as precise control over the treatment location and timing, localized healing without affecting surrounding healthy tissues, and deep penetration into tissues reaching deeper wound layers that are otherwise challenging to treat. Moreover, it is shown that NIR light stimulates cellular processes that promote wound healing, such as increased blood flow, collagen synthesis, and cell proliferation [26,27,28]. Studies have investigated using NIR light therapy for various wounds, including diabetic foot ulcers, pressure ulcers, and surgical wounds, where it is shown that patients who received NIR light therapy had faster wound healing rates and lower infection rates than those who did not receive the treatment [26,28,29,30]. The synergy between NPs and NIR light leverages the advantages of both modalities, making it a promising tool for advanced wound care. For example, some studies show that NIR laser-excited triangular AgNPs can effectively kill bacteria and accelerate wound closure [31]. Khan et al. used IR photo-absorbing graphene oxide to generate heat from laser energy for antibacterial and antifungal wound infection control [32].

Moreover, a study by Kang et al. reveals the effects of NIR irradiation on the enhancement of collagen production and activation of the human keratinocyte and dermal fibroblast proliferation through NIR-emitting Ag_2_S NPs [33]. Also, other studies have investigated nanofibers coated with biocompatible IR-absorbing NPs with NIR-mediated photothermal effects that efficiently prevent bacterial infections and can be applied directly to wounds to promote healing. All of the above signifies the enormous clinical potential of photodynamic therapy and nanotechnology in wound-care management.

Following the idea to develop the desired system that can kill microorganisms and simultaneously stimulate skin regeneration, we have fabricated hybrid materials made of AgMOF containing S- or N-carbon dots (CDs) (AgMOFS-CDs NPs or AgMOFN-CDs NPs, respectively) to leverage all the aforementioned benefits of the individual components to achieve enhanced features. These hybrid NPs were previously characterized and showed antibacterial properties comparable or superior to some antibiotics on Gram-negative (*E. coli*) and Gram-positive (*B. suptilis*) bacteria [34]. The formation of the hybrid NPs involved linking the sulfonic groups, sulfates, amines, amides, and carboxyl groups present in the S- and N-containing CDs to the silver centers of the AgMOF component. This association led to the formation of rod-like NPs with perforated surfaces and smaller size silver oxide particles inside hybrid NPs than in parent AgMOF. Moreover, an association of the N-CDs with AgMOFs resulted in a charge transfer process, which resulted in more mobile silver ions and a more positive charge to the AgMOFN-CDs. All these characteristics of hybrid NPs resulted in facilitated electrostatic interactions with bacterial cell membranes and improved antibacterial activity compared to individual components. These enhanced antibacterial properties, high porosity, and large surface area, alongside MOFs’ mechanical strength that makes them suitable for incorporation into wound dressings that require durability and flexibility, can be leveraged to design advanced wound care materials that deliver drugs, prevent infections, and promote the natural healing processes. Our study was designed to elucidate the potential of these unique hybrid materials combined with NIR light for wound healing and point to some underlying mechanisms related to cell proliferation and migration, the production of extracellular matrices, immunomodulatory effects, and protein structural changes.

## 2. Materials and Methods

### 2.1. Materials

Ag-1,3,5-benzene tricarboxylic acid MOFs with S- and N-(CDs) were synthesized and characterized as described previously [34]. Trichloroacetic acid, sulforhodamine B (SRB), acetic acid, and TRIS buffer were purchased from Sigma-Aldrich, St. Louis, MO, USA.

### 2.2. Cell Culture

Human fibroblasts (MRC-5) (ATCC, Rockville, MD, USA) and keratinocytes (HaCaT) (Cell Line Service GmbH, Eppelheim, Germany) were cultured in Dulbecco’s Modified Eagle’s Medium DMEM (Capricorn Scientific GmbH, Ebsdorfergrund-Dreihausen, Germany) supplemented with 10% fetal bovine serum (FBS), 100 units/mL penicillin, and 100 μg/mL streptomycin (all from Thermo Fisher Scientific, Waltham, MA, USA). The cells were maintained at 37 °C in a humidified chamber with 5% CO_2_ (ESCO, Lifesciences Group, Singapore).

### 2.3. Assessment of Cell Viability

Cells were seeded in 96-well plates. After 24 h, they were treated with AgMOFS-CDs NPs and AgMOFN-CDs NPs with various concentrations of 0.00125 mg/mL, 0.0006 mg/mL, 0.0003 mg/mL, 0.00015 mg/mL, and 0.000075 mg/mL for 48 h. After that period, an SRB assay was performed for cell viability determination based on the measurement of cellular protein content as described in the literature [35]. The optical density at a wavelength of 550 nm was measured by a microplate reader (Wallac, VICTOR2 1420 Multilabel counter, PerkinElmer, Turku, Finland).

### 2.4. Laser Illumination

The light beam (2 mm wide, input power 520 mW, pulse width 42 fs, central wavelength 850 nm) from a mode-locked Titanium: Sapphire laser system (Mantis, Coherent, Santa Clara, CA, USA) in continuous-wave regime was split into two beams by a non-polarizing beam-splitter cube (BS011, Thorlabs, Newton, NJ, USA). These beams were uniformly expanded by two pairs of UV-fused spherical plano-concave and plano-convex uncoated lenses (LC4291 and LA4725, Thorlabs) and reflected from broadband dielectric mirrors (BB1-EO3, Thorlabs) onto wells of microtiter plate in which either keratinocytes or fibroblasts cells were seeded. Where necessary, depending on the size of wells, the beams were additionally expanded by plano-concave UV-fused spherical uncoated lenses (LC4513, Thorlabs). Stray light was eliminated by post-mountable iris diaphragms (ID25/M, Thorlabs), whereas the beams’ intensities were controlled by continuously variable metallic neutral density filters (NDL-10C-4, Thorlabs). The illumination duration was 3, 5, or 8 min, whereas light intensity above the microtiter plate was 8, 16, or 60 mW/cm^2^. The intensity was measured at 850 nm by Nova II (Ophir, Jerusalem, Israel) laser power meter with a PD300-3W sensor (Ophir). Additionally, to minimize the thermal stress of cells, we have kept the bottom of microtiter plates at 37 °C.

### 2.5. Immunofluorescence Staining for Determination of Ki-67

The proliferation index was assessed by applying Ki-67 immunofluorescent staining. Cells were grown on polylysine-coated slides (Merck, Darmstadt, Germany) and left to adhere for 24 h, upon which they were treated for 48 h, as previously described [36]. After the treatment, the cells were processed according to the standard procedures (fixed in 4% formaldehyde solution, permeabilized with 0.25% Triton-X (Merck), blocked with 1% BSA solution), and stained with Ki-67 Monoclonal Antibody SP6 (MA5-14520, Thermo Fisher Scientific) at 1:250 dilution overnight at 4 °C. First, the slides were washed in 1 × phosphate-buffered saline (PBS) solution. Next, goat anti-rabbit IgG (H + L) cross-adsorbed secondary antibody, Cyanine3 (A10520, Thermo Fisher Scientific) at the final concentration of 10 µg/mL was applied for two hours at room temperature, upon which the slides were washed in 1 × PBS solution, dehydrated in series of ethanol solutions (70%, 90%, and absolute), and stained using DAPI-Vectashield solution (Vector laboratories, Newark, CA, USA). At least 500 cells per treatment were analyzed using the Zeiss-Axioimager A1 microscope (Carl Zeiss, Jena, Germany) and the ISIS imaging software package version 5.8 (MetaSystems, Altlussheim, Germany), and the proliferation index (PI) was expressed as the ratio between Ki-67+ cells and the total number of cells. The results were presented as means ± standard error of the mean.

### 2.6. Hydroxyproline Assay

After the treatments, cells were collected and lysed by freeze-thawing, and the protein concentration and hydroxyproline assay were performed as described elsewhere [37,38]. Proteins in each standard (collagen from calf skin) and cell lysate samples were hydrolyzed to amino acids by adding equal volumes of 4 N sodium hydroxide (NaOH) and 1 h incubation at 120 °C. Samples were allowed to cool to room temperature and then neutralized with the same volume of 4 N hydrochloric acid (HCl). Hydroxyproline was converted to pyrrole-2-carboxylate by oxidation via the addition of chloramine-T solution (0.05 M in 74% *v*/*v* H_2_O, 26% *v*/*v* 2-propanol, 0.629 M NaOH, 0.140 M citric acid, 0.453 M sodium acetate, and 0.112 M glacial acetic acid, 20 min, room temperature). Afterwards, Ehrlich’s solution was added to each sample, vortexed, incubated at 65 °C for 20 min, and rapidly cooled to stop chromophore development. Optical densities were detected by a spectrophotometer at 550 nm. Values were calculated as equivalents of the collagen standard curve and normalized per mg of proteins in the samples. Results were presented as a % of hydroxyproline content relative to control.

### 2.7. Interleukin-6 and Interleukin-1 Assay

For IL-1β determination in samples, an ELISA kit from R&D Systems (Minneapolis, MN, USA) was employed (Cat. No. Dy201) with a detection range of 3.9 pg/mL–250 pg/mL. Both cytokines were quantified according to the manufacturer’s instructions for the respective duo-set kits. Briefly, test samples were thawed for 1 h at 37 °C, vortexed, and centrifuged at 300× *g* for 10 min, and supernatants were used for the assay. ELISA plates (high binding, Corning, Glendale, AZ, USA) were coated overnight at room temperature with “capture” antibodies for IL-1β (4 µg/mL), then blocked with 1% BSA in PBS for 1 h. Samples and standards were incubated at room temperature for 2 h, followed by biotinylated antibodies for IL-1β (200 ng/mL) for 2 h at room temperature. Afterwards, samples were incubated with streptavidin-horseradish peroxidase for 20 min. The peroxidase substrate was added, and the OD was measured at 450 (reference reading correction was 570 nm). The absorbances obtained for each standard and sample were adjusted: OD = OD450 − OD570. Standard curves and sample concentrations were calculated using GraphPad Prism 8.1 software (GraphPad Software Inc., Dotmatics, Boston, MA, USA) employing a 4-parameter sigmoidal curve fitting.

### 2.8. Migration (Scratch) Assay

Fibroblast cells were seeded in 6-well plates (5 × 10^5^ per well). Wounds were made mechanically with a sterile 200 μL tip, the medium was disposed of, and wells were washed with PBS to remove all detached cells. Next, fresh cell medium without FCS was added, and images were acquired through a Zeiss Axiovert 200 microscope (Carl Zeiss, Oberkochen, Germany) using a 10× objective connected to an AxioCAM camera. Subsequently, cells were treated, illuminated, or both, then incubated at 37 °C with 5% CO_2_. Images were acquired again after 24 and 48 h of incubation without disposing of the media. Image J software (version 1.53t, National Institutes of Health, Bethesda, MD, USA) was used with the plugin wound healing size tool [39] to measure the scratched area.

Wound closure is calculated according to the following equation:

wound closure (%) = ((W_0_ − W_t_)/W_0_) × 100,

where W_0_ = wound area at 0 h and W_t_ = wound area at ∆h.

### 2.9. SR-FTIR Spectroscopy

Fibroblast cells were grown on CaF_2_ glass in a 12-well plate and treated with AgMOFN-CDs and AgMOFS-CDs NPs (0.0003 mg/mL) in the dark and upon light illumination (16 mW/cm^2^, 3 min). After incubation for 24 h, the cells were rinsed with physiological solution (0.9% NaCl) three times and fixed with 4% formaldehyde for 15 min. After discarded formaldehyde, the cells were dehydrated by successive washing with increasing ethanol concentrations (70%, 95%, and absolute ethanol, 5 min each step), followed by drying at room temperature. Cells were stored in a desiccator until FTIR measurements.

The structural analysis of proteins in MRC-5 cells before and after exposure to AgMOFN-CDs, AgMOFS-CDs, NIR illumination, and combined treatments was conducted using SR-FTIR at the Synchrotron ALBA at the MIRAS beamline, located in Barcelona, Spain, utilizing synchrotron radiation for the IR source. A Hyperion 3000 microscope (Bruker, Billerica, MA, USA) attached to a Vertex 70 v spectrometer (Bruker) was employed, with a mercury cadmium telluride MCT detector cooled by liquid nitrogen. The FTIR microscope’s aperture was adjusted to the dimensions of a single cell (12 × 12 µm^2^), analyzing 30 cells per treatment group. The acquisition of spectroscopic data was in transmission mode, employing a 36× Schwarzschild objective (Newport, Irvine, CA, USA) and condenser across the mid-IR spectrum of 4000–900 cm^−1^.

### 2.10. Optical Properties

A UV-Vis spectrophotometer (Shimadzu, Kyoto, Japan) was used to record the UV-Vis spectra of the AgMOFS-CDs and AgMOFN-CDs. The spectra of the hybrid materials were recorded from 200 to 1200 nm with H_2_O/DMSO (4: 1, *v*/*v*) as a blank. The potential presence of a photothermal effect was examined by type K thermometer Testo 925 (Testo, West Chester, PA, USA), whose flexible immersion tip has been carefully plunged into a corresponding well filled with seeded cells and DMEM supplemented with FBS. Experiments were performed in the dark at constant room temperature. The emission spectra of saline solution, AgMOFS-CDs, and AgMOFN-CDs have been taken by USB 2000+ fiber optic spectrometer (Ocean Optics, Dunedin, FL, USA).

### 2.11. Data Processing and Statistical Analysis

Cell viability results were analyzed by one-way analysis of variance (ANOVA) and in GraphPad Prism 8.0 program (GraphPadSoftware, San Diego, CA, USA). Differences of *p* ≤ 0.05 were considered significant. Data were expressed as the percentage of cell viability normalized to control. Each data point represents the mean ± standard deviation (SD) of quadruplicate determinations.

For the proliferation index, statistical analysis was performed using SPSS 10 for Windows (IBM, Armonk, NY, USA) and a one-way ANOVA statistical test. *p* values < 0.05 were defined as the level of significance. Experiments were performed in triplicates.

For SR FTIR data processing and statistical analysis, the OPUS 8.2 software (Bruker) and the Quasar software package version 1.5.0 (Bioinformatics Laboratory at the University of Ljubljana, Ljubljana, Slovenia) were used, respectively. Before principal component analysis (PCA), the region of Amides I and II, i.e., 1720–1480 cm^−1^ of the FTIR spectra, were baseline-corrected via the rubber band method and vector-normalized. The second derivative of spectra was calculated by using the Savitzky–Golay algorithm (window 11, third polynomial order, derivative order 2). In total, 30 individual cells for each study group were selected using the microscope.

## 3. Results

### 3.1. Synergic Effect of Laser Illumination and AgMOF-CDs Treatments

**Figure 1:**
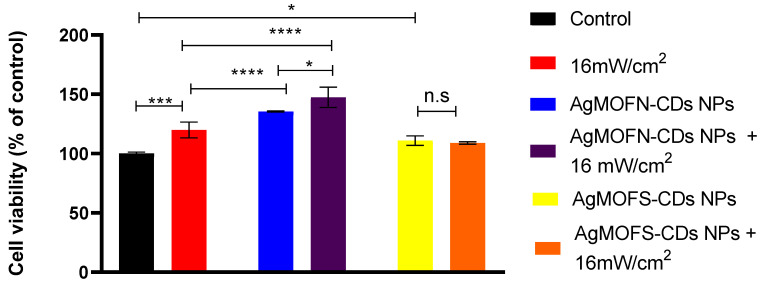
Effects of laser illumination and treatment with AgMOF-CDs NPs on fibroblast cell viability assessed by SRB assay. The cells were illuminated with a laser intensity of 16 mW/cm^2^ for 3 min without NPs or with AgMOFN-CDs NPs or AgMOFS-CDs NPs (0.0003 mg/mL). Before NIR light illumination, cells were treated with AgMOF NP for 3 h, and the cell viability was determined 48 h after the illumination. * *p* < 0.05; *** *p* < 0.001; **** *p* < 0.0001. n.s.—not significant.

In the first phase, optimum treatment parameters for cell growth were studied. Based on the results detailed in the Supplementary Materials (Figures S1–S6), the most favorable conditions for stimulating cell growth was a 3 min laser illumination at an intensity of 16 mW/cm^2^ in combination with 0.0003 mg/mL of AgMOFN-CDs NPs or AgMOFS-CDs NPs. These conditions were selected for further investigation, and the light/NP synergistic effect on fibroblasts’ cell viability is given in Figure 1. During each illumination experiment, the system’s temperature was measured, and no increase in the temperature was detected.

Cell viability was increased by treating the cells with AgMOFN-CDs (140% cell viability compared to control) and AgMOFS-CDs (110% compared to untreated fibroblasts). NIR light illumination of AgMOFS-CDs-treated cells did not additionally increase the cell viability. In contrast, in the case of AgMOFN-CDs-treated cells, a significant rise in cell viability was detected (cell viability rise to 150%). A similar effect was observed with keratocytes (Figure S7).

### 3.2. Optical Properties and Mechanism of Action of Hybrid Nanoparticles

**Figure 2:**
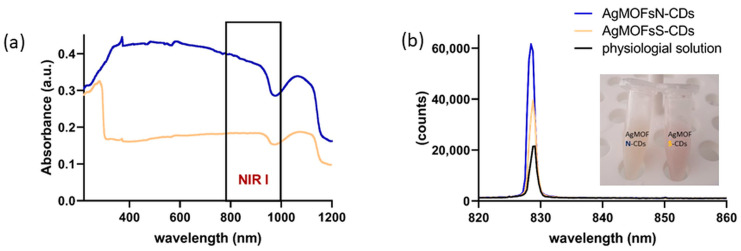
(**a**) UV-Vis-NIR absorption spectrum of AgMOFS-CDs and AgMOFN-CDs solutions, showing a strong AgMOFN-CDs absorbance band in the NIR region. (**b**) Emission capacity of the control (physiological solution), AgMOFS-CDs, and AgMOFN-CDs. Solutions of the AgMOFS-CDs and AgMOFN-CDs hybrid materials are given in insertion in (**b**).

The UV-Vis-NIR spectrum of NPs (Figure 2a) was recorded to investigate the light absorption performance of hybrid materials. As shown in Figure 2a, the AgMOFN-CDs sample exhibited a wide absorbing spectral range from UV, visible to the NIR region, while AgMOFS-CDs show the highest absorption in the UV range.

The light emission (Figure 2b) was recorded under the same illumination conditions as in experiments with cells to check if the light emission might contribute to the observed cellular effects. We acquired the emission of the solvent (physiological solution) as a control and solutions of AgMOFN-CDs and AgMOFS-CDs after 3 min of NIR laser illumination (16 mW/cm^2^). The results presented in Figure 2b show that the illumination of AgMOFS-CDs was double the emission of the control solution, whereas AgMOFN-CDs showed three times higher emission than the control. The emission of AgMOFN-CDs was 1.6 times higher than that of AgMOFS-CDs, which is in good correlation with the absorption characteristics of hybrid materials (Figure 2a).

### 3.3. Fibrobroblasts’ Proliferation

**Figure 3:**
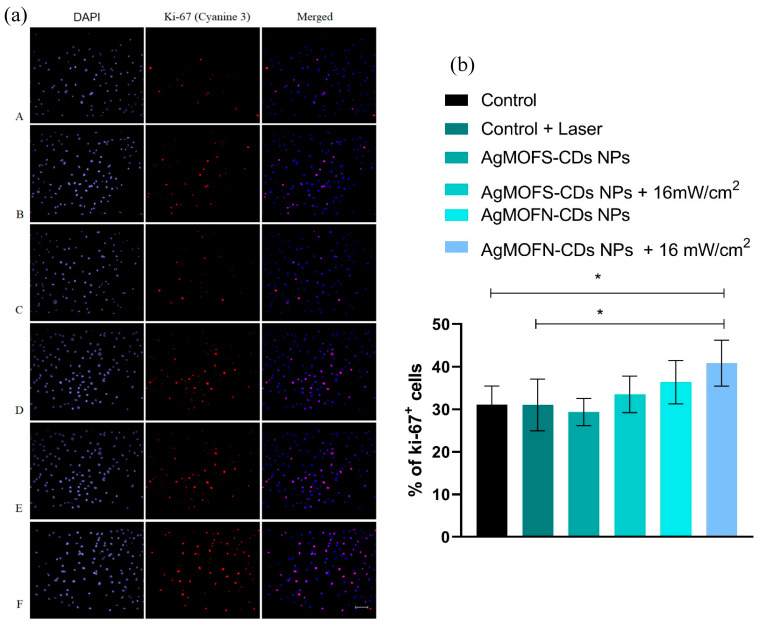
(**a**) Ki-67 immunofluorescent staining of control (untreated) MRC-5 cells (**A**); laser-illuminated cells (**B**), AgMOFS-CDs NPs-treated cells (**C**), AgMOFS-CDs NPs-treated and laser-illuminated cells (**D**); AgMOFN-CDs NPs-treated cells (**E**), AgMOFN-CDs NPs-treated and laser-illuminated cells (**F**). The left panel shows DAPI-stained cells; the middle panel (Cyanine 3) shows proliferative active Ki-67^+^ cells; the right panel shows merged DAPI + Cyanine 3 photomicrographs. Scale bar = 100 μm. (**b**) The values of proliferation index of fibroblast cells treated with AgMOFS-CDs and AgMOFN-CDs NPs without and with a laser intensity of 16 mW/cm^2^ within 3 min. (* *p* < 0.05).

Fibroblasts’ proliferation was assessed through the level of Ki-67 protein expression in untreated and NPs/light-treated cells. The percentage of Ki-67^+^ cells in the population gives the proliferation index (the percentage of dividing cells in the culture). The proliferation of fibroblasts in the dark remained at the control level in the case of AgMOFS-CDs NPs treatment. In contrast, in the AgMOFN-CDs treatment, the proliferation increased but did not differ significantly from the control level (Figure 3a,b). When the cells are illuminated after the treatment with NPs, proliferation increases; this increase was insignificant when the cells were previously treated with AgMOFS-CDs NPs. Still, the proliferation was significant when the cells were treated with AgMOFN-CDs NPs/NIR light combination (*p* < 0.05).

### 3.4. Effect of NPs/Light Treatment on Collagen Production

Augmented metabolic activity of fibroblasts after the NPs/light treatment of fibroblasts is reflected by the de-novo synthesis of collagen as an essential component of the extracellular matrix, which was reflected by a higher concentration of hydroxyproline after the treatments. The level of detected hydroxyproline was 5% higher than the control after treatment with AgMOFN-CDs NPs and 10% after combined AgMOFN-CDs NPs/light treatment (Figure S8). Although insignificant, the detected increase in hydroxyproline concentrations 48 h after treatment indicates a positive outcome.

### 3.5. Effect of NPs/Light Treatment on Fibroblasts Migration

**Figure 4:**
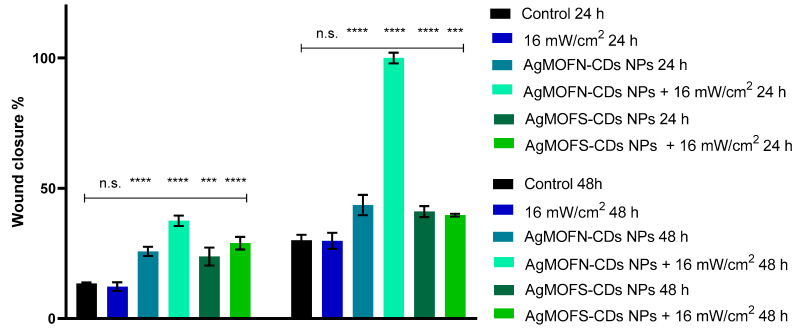
Percentage of wound closure monitored at 24 and 48 h after the indicated treatment. (n.s. not significant; *** *p* < 0.001; **** *p* < 0.0001).

**Figure 5:**
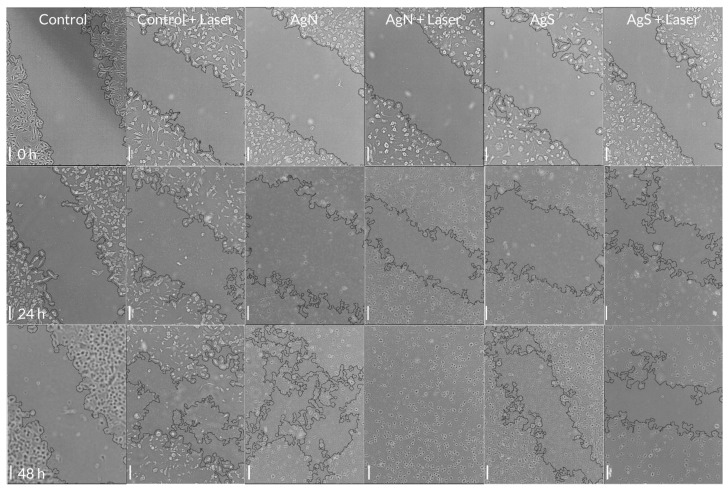
Photomicrographs of cell culture model of wound healing (fibroblast cells). Images on the first row are captured at 0 h, the second after 24 h of treatment, and the third after 48 h. The first column represents images of untreated cells, the second illuminated with a laser intensity of 16 mW/cm^2^, the third treated with 0.0003 mg/mL AgN (AgMOFN-CDs), the fourth AgN (AgMOFN-CDs) + laser illumination of 16 mW/cm^2^; the fifth column represents images of cells treated with 0.0003 mg/mL AgS (AgMOFS-CDs), and the sixth column AgS (AgMOFS-CDs) + laser illumination of 16 mW/cm^2^. Scale bar = 100 μm.

Migration or “scratch” assay performed on fibroblasts showed no significant effect of NIR light illumination without previous treatment (Figure 4 and Figure 5). The treatment of fibroblasts with AgMOFS-CDs in the dark significantly facilitated cell migration at both time points compared to the control. A combination of NIR light and AgMOFS-CDs temporarily enhanced this effect at 24 h, with no pronounced difference observed at 48 h.

The combination of AgMOFN-CDs treatment and NIR illumination exhibited the most significant enhancement in cell migration, achieving almost a complete wound closure at 48 h, Figure 5. This effect was statistically significant compared to the impact of AgMOFN-CDs treatment in the dark (Figure 4), indicating that the synergistic interaction between AgMOFN-CDs and NIR illumination stimulates cell migration.

### 3.6. Effect of NPs/Light Treatment on IL-1 Levels

To elucidate the production of pro-inflammatory cytokines, which play a crucial role in regulating immune cell functions during the healing process, we have measured levels of IL-1 and IL-6 after the fibroblast treatment. All tested values for IL-6 were below the sensitivity threshold of the detection kit. As indicated in Table 1, the IL-1 level increased after the treatment with AgMOFS-CDs compared to the control. In contrast, there was a significantly lower IL-1 expression in the AgMOFN-CDs-treated sample compared with the control group. In contrast, a combined treatment with light decreased IL-1 values compared to unilluminated analogous.

### 3.7. Structural Alterations in Fibroblasts Proteins Obtained by SR-FTIR Spectroscopy

**Figure 6:**
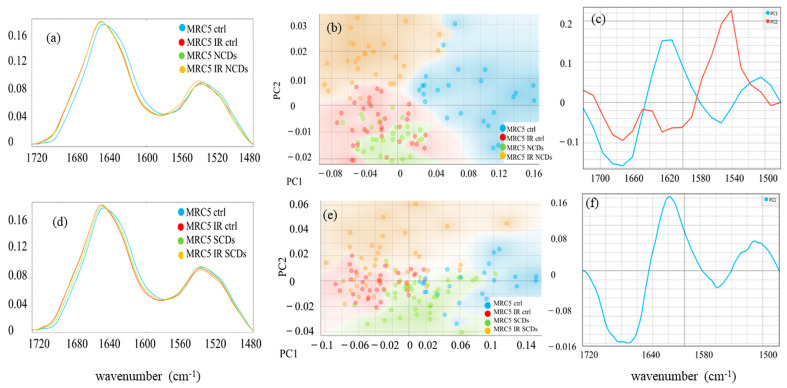
The SR-FTIR spectra of Amide I and Amide II regions, including the ester groups (1480–1800 cm^−1^) in two sets of experiments with AgMOFsN-CDs (NCDs) (**a**) and AgMOFsS-CDs (SCDs) (**d**) and corresponding PCA scores plot (**b**,**e**), PCA loadings profile (**c**,**f**) of the protein and ester regions (1500–1700 cm^−1^) of the untreated control cells (blue line) and cells treated with AgMOFN-CDs or AgMOFS-CDs (green line), illuminated with NIR light (red line), and treated and illuminated cells (combined treatment, orange line).

SR FTIR spectra of immobilized control and NP/light-treated cells were analyzed to elucidate structural changes of proteins. The protein fingerprint region encompasses the wavenumbers between 1480 and around 1800 cm^−1^. It contains two major vibrations: Amide I (associated with the C=O stretching vibration of the peptide backbone) and Amide II bands (N-H bending vibrations). These spectra are shown in Figure 6a,d.

It is apparent from Figure 6a,d that the changes induced by AgMOFsN-CDs alone and in combination with NIR light are more pronounced than those observed in the AgMOFsS-CDs-tested group. The signal corresponding to the Amide I region shifts from 1652 cm^−1^ to 1656 cm^−1^ in all treated samples (Figure 6a). On the other hand, the Amide II band shift from 1538 cm^−1^ to 1542 cm^−1^ was observed only in the AgMOFN-CDs/light-treated cells.

In the test group treated with AgMOFS-CDs, Figure 6d shows a mild shift of the Amide I band to higher wavenumbers compared to the control group, which was induced by AgMOFS-CDs in the dark (green line). However, a more pronounced shift of the same peak (from 1652 cm^−1^ to 1655 cm^−1^) was observed following illumination. The application of NIR light, both independently and in conjunction with AgMOFS-CDs, induced similar protein structure alterations.

We have used PCA to compare FTIR spectral data between control and treated cells, highlighting key protein differences. In the experiments with AgMOFN-CDs, the PCA scatter plot reveals distinct clustering patterns and groupings among control cells and those subjected to various treatments (Figure 6b). The control groups are clustered mainly on the positive side of the PC1 axis, whereas all treated groups are clustered on the negative side of the PC-1 axis. The separation along the PC1 axis underscores the significant alterations in the proteins upon treatment, either with AgMOFN-CDs, NIR illumination, or their combination. The PC1 loading plot was utilized to identify protein bands that were significant discriminators between control and treated cells, highlighting the critical spectral differences driven by treatment. In the PC1 loading plot, Figure 6c, the minimums at 1556 cm^−1^ (N-H bending vibration) and 1680 cm^−1^ (antiparallel β-sheet and turn) [40] along with the maximums at 1510 cm^−1^ (tyrosine (Tyr) side chain)) [41] and 1620 cm^−1^ (cross-β) [40] emerge as critical discriminators between control and treated cells. All treated samples exhibit a relative increase in N-H bending vibrations and antiparallel β-sheet structures and a decrease in the Tyr side chain and cross-β content compared to controls. The clustering of cells treated with AgMOFN-CDs and those illuminated with NIR light in the same region (negative PC2) suggests that both treatments induce similar biochemical changes within the cells, possibly affecting analogous molecular pathways or structures.

Conversely, the unique positioning of data sets subjected to the combined treatment (AgMOFN-CDs and NIR light) in the positive region of PC2 (Figure 6c, orange cluster) is observed, separating them distinctly from cells receiving individual treatments. The distinct separation over the PC2 axis between cells subjected to combined treatments versus those with individual treatments underscores the unique biochemical or structural changes induced by the synergistic or additive effects of AgMOFN-CDs and NIR light. Specifically, the increased prominence of α-helix structures in the combined treatment group (maximums PC2 at 1647 and 1542 cm^−1^ in loading plot Figure 6c) indicates that the combined treatment uniquely alters the protein landscape beyond the alterations of individual treatments.

The effect of AgMOFS-CDs was similar to AgMOFsN-CDs, though it had a lower impact (Figure 6d–f). The PCA scatter plot in the set of experiments with AgMOFS-CDs, Figure 6e, showed a distinct separation along the PC1 axis between control samples (positive side of PC1 axis) and those subjected to NIR illumination and combined treatment (negative side of PC1), highlighting apparent differences in response. However, unlike experiments with AgMOFN-CDs, treatments with AgMOFS-CDs showed dispersed distribution across the PC1 axis and less distinct clustering and differentiation between NIR and combined treatments. The separation over the PC2 axis was not detected, and therefore, it was not shown in Figure 6f.

**Figure 7:**
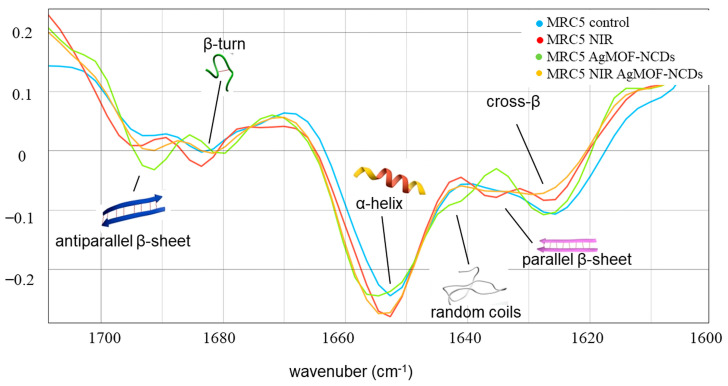
The second derivative of the Amide I band (1500–1700 cm^−1^) of the untreated control cells (blue line) and cells treated with AgMOFN-CDS (green line), illuminated with NIR light (red line), and AgMOFN-CDs-treated and illuminated cells (combined treatment, orange line) with indicated secondary structures.

To obtain more detailed information about protein conformational changes more significantly induced by AgMOFN-CDs and NIR illumination, Amide I band analysis, susceptible to secondary structure alterations, was conducted using second-derivative spectra refined with the Savitzky–Golay algorithm (nine smoothing points, third polynomial order) and vector normalization, Figure 7. The band assignments based on the established literature are presented in Table 2 [40,42]. The second-derivative of the Amide I band (1600–1700 cm^−1^) in control fibroblast cells revealed that this band consists of the following components: antiparallel β-sheets as well as turn and loops (peaks at ~1692 and 1682 ± 2 cm^−1^), α-helices (1653 ± 2 cm^−1^), random coils (1642 cm^−1^), parallel β-sheet (1638 cm^−1^), and cross-β (1627 cm^−1^). Following the treatments, the observed changes include alterations in the intensity of secondary structures, indicating a change in their content, and the emergence of new peaks, suggesting the formation of new secondary structures alongside broadening existing bands, reflecting increased structural diversity or heterogeneity.

The shift of the α-helix peak after AgMOFN-CDs treatment, together with the appearance of two new signals at 1692 cm^−1^ and 1642 cm^−1^, assigned to antiparallel β-sheet and random coils, respectively, suggests that AgMOFN-CDs induce a disruption or unfolding of more organized structures, i.e., the destabilization of α-helices, promoting antiparallel β-sheet and random coil configurations, which could affect protein function and interactions. NIR light exposure to fibroblasts induces an increase in α-helices and β-turn, as indicated in Figure 7.

The combined treatment with NIR and AgMOFN-CDs creates an intermediate effect and reduces cross-β content while increasing α-helix content. Not only enhancing the α-helical content (as indicated by the increased intensity) was observed, but also introducing a broader range of helix conformations (evidenced by the broadening of the peak, Figure 7), and inducing a subtle shift (from 1652 to 1654 cm^−1^), reflecting a structural modification.

**Figure 8:**
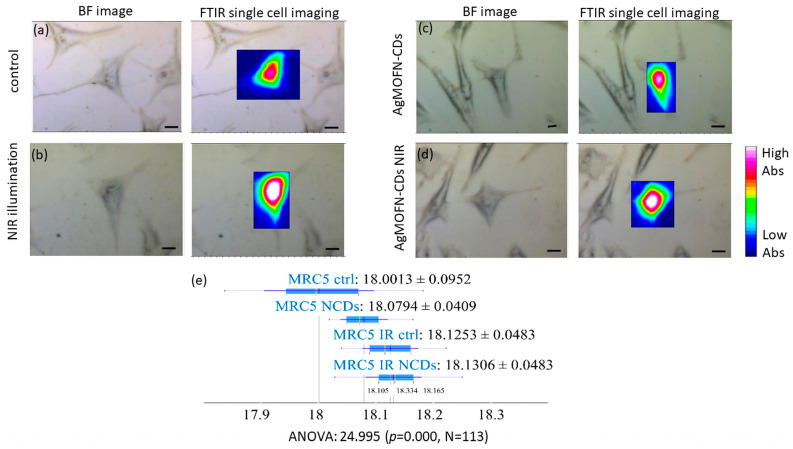
SR-FTIR imaging. Visible and FTIR images based on the integration of Amide I and II bands which were pre-processed using baseline correction and normalization. Scale bars: 10 µm. A single MRC-5 control cell (**a**). A single MRC-5 cell illuminated with NIR light (**b**), treated with AgMOFN-CDs (**c**), illuminated and treated with AgMOFN-CDs (combined treatment) (**d**). Scaling of the contour colors has been applied for the bands, with blue/light pink equivalent to low/high absorption intensity. Total protein content in control and treated fibroblast cells generated based on Amide I and II band integrations (1700–1480 cm^−1^) (**e**).

We have also assessed the total protein content in the control and treated fibroblast cells generated based on Amide I and II band integrations (1700–1480 cm^−1^) (Figure 8e) and chemical mapping imaging of single cells to compare protein localization in control and treated cells (Figure 8a–d). The results indicate a higher content of proteins and their altered spatial distribution in the central part of the cells in cells treated and/or illuminated with NIR light compared to the control (Figure 8e). There was an observed increase in protein content across all treated samples, with the highest values detected in samples subjected to AgMOFN-CDs/light treatment and NIR illumination.

## 4. Discussion

The ideal wound healing system should possess inherent antibacterial characteristics, ensuring effective bacterial inhibition and exhibiting enhanced wound closure by promoting cell proliferation and synthesis of extracellular matrices. The previous work demonstrated the antibacterial effect of AgMOFN-CDs and AgMOFS-CDs NPs [34]. Among these two tested analogues, AgMOFN-CDs stood out as the more effective antibacterial agents against both Gram (+) (*B. subtilis*) and Gram (−) (*E. coli*) bacteria. AgMOFN-CDs inhibit *E. coli* growth by 90% at the concentration 4 µg/mL, which is significantly lower compared to traditional antibiotics like ampicillin, florfenicol, neomycin, and spectinomycin, which have minimal inhibition concentration values of >32.0, 8.0, 512.0, and >128.0 µg/mL, respectively. The authors also showed that the antibacterial activity of AgMOFN-CDs at the concentration of 0.5 µg/mL notably inhibits the *B. subtilis* growth by nearly 20%. On the other hand, in our work, we have demonstrated that a concentration of 0.4 µg/mL stimulates fibroblast cell growth by 40%, which was additionally enhanced by NIR light illumination, and that higher concentrations up to 2 µg/mL do not significantly affect cell viability. From the treatment point of view, a 4 µg/mL concentration can be used to control bacterial infections, and once the infection is managed, the dosage can be reduced to 0.4 µg/mL in combination with NIR PDT to specifically promote fibroblast growth and enhance healing. Such a strategy allows for a tailored treatment approach, adjusting the AgMOFsN-CDs concentration based on the healing stage and infection status. This dual-functional capability supports the chemical’s development as a versatile and effective agent in clinical settings, particularly for managing complex wound environments. Therefore, in this work, we tested the potential of combining those NPs with NIR light to promote wound healing and tackle the underlying mechanisms. Our results strongly support the synergistic effect of NIR light on the wound healing of those previously treated with AgMOFN-CDs and the system’s potential to be applied in clinical studies.

Based on the results obtained with keratinocytes, which are given in the Supplementary Materials, we can speculate that the observed effect was universal for the wound healing process. However, because of the pivotal role of fibroblasts in wound healing, covering everything from extracellular matrix component deposition and wound contraction to managing inflammation and scarring, [43,44] our study focused more on healing mechanisms induced in fibroblasts.

A combination of AgMOFN-CDs and NIR light resulted in a completely closed wound area after 48 h, induced by the scratch of the fibroblast cell culture (Figure 4 and Figure 5). Although specific literature data suggest that photo biomodulation promotes cell migration [27,45], our results indicated that applied NIR light illumination alone did not significantly enhance cell migration compared to the control group at 24 and 48 h, Figure 4 and Figure 5, at least under the applied experimental conditions. This result suggests that without additional treatments, NIR light does not play a critical role in modulating fibroblast migration and that AgMOFN-CDs mediate significant effects. However, a synergistic effect could be observed. This result is consistent with other impacts of the AgMOFN-CDs/light treatment, i.e., the stimulation cell viability (Figure 1), detected proliferation index (Figure 3) and collagen production measured by the concentration of hydroxyproline in the medium (Figure S8). Other authors have shown that the illumination of fibroblasts by red or infrared light increases the production of extracellular matrix proteins, such as elastin, collagen, and hyaluronic acid [46]. Although statistically non-significant, our results show a slight increase in collagen production after the NPs/light treatment, which is consistent with the migration/wound closing assay. However, the experimental conditions are not comparable to those applied by Kim and co-workers in their work, as we have delivered to cells a higher intensity in a single dosage. Although we have not determined the production of other extracellular matrix components, slightly increased collagen production might explain the increased cell migration upon the NPs/light treatment.

A transient effect of AgMOFS-CDs/light on cell migration is obtained (Figure 4 and Figure 5), suggesting that AgMOFS-CDs-NPs/light initially boosts the healing process, which is not sustained over time and does not result in a complete wound closure. Indeed, this effect may be more expressed if the light treatment is repeated, as obtained by Kim and co-workers [46]. Still, our goal was to trigger intracellular processes, leading to wound healing/closure in a quick and efficient manner. Therefore, although repeated exposure of skin wounds to NIR light triggers tissue regeneration, previous treatment with AgMOFN-CDs is less tedious since the process might be completed quickly.

Judging by the expression of Ki-67 after the treatments, the fibroblast proliferation also confirms that the AgMOFN-CDs/light combination is the most efficient in stimulating cell proliferation. Ki-67 is a nuclear protein expressed in all dividing cells through all cell cycle phases (G1, S, G2, and M) except in the G0 phase. Even though it is mainly used in diagnostics to assess tumor progression and response to therapy, it was demonstrated that this protein is required for healthy cell–cell cycle progression and DNA replication [47]. A dual synergistic increase in fibroblast proliferation and cell viability observed uniquely with the combined treatment with AgMOFN-CDS and NIR illumination suggests that the combined treatment promotes both the survival and division of cells, indicating activation of specific cellular pathways or mechanisms that are not as effectively triggered by individual treatments.

Balanced immune responses facilitate effective wound healing, as optimal pro-inflammatory cytokine levels prevent infections and expedite natural healing. Nevertheless, when the immune system becomes dysregulated during wound healing, which is characteristic of chronic wounds, it can result in prolonged inflammation and delayed healing [43]. Current studies implicated that the microenvironment of a chronic wound, especially in diabetic ulcers, is characterized by overexpression of inflammatory mediators such as IL-1β, which further mediates delayed wound healing in diabetic patients by reducing cell proliferation and migration [43,44]. Dai et al. observed an inhibitory effect on fibroblast proliferation and migration under high concentrations of IL-1β, whereas low concentrations of IL-1β promoted cell proliferation [44]. Furthermore, they uncovered that introducing IL-1β led to the activation of MMP expression and the suppression of tissue inhibitors of metalloproteinases (TIMPs) and collagenase production. Furuyama et al. showed that the secretion of matrix metalloproteinases MMP-2 and MMP-9 in fibroblasts, induced by IL-1β, suppresses basement membrane formation [45]. This result indicates IL-1β’s involvement in enhancing extracellular matrix degradation and extending the delay in the wound healing process among diabetes patients. Therefore, inhibiting these cytokines or reducing their level with drugs may be one of the mechanisms to modulate the inflammatory reaction in chronic wounds and promote better wound healing. Considering the importance of these molecules in the wound healing process, we explore the impact of NPs treatment with and without light exposure on the levels of IL-1. The most significant reduction in IL-1 level was observed in combined treatment AgMOFN-CDs with NIR light, implying that this treatment could potentially address the excessive inflammation seen in conditions like diabetic wounds and create a more conducive environment for healing. We conclude that treatment with AgMOFN-CDs, especially in combination with light, can modulate local inflammatory responses, promote fibroblast migration, and induce a slight increase in collagen in the cells. A relation between reduced IL-1 levels and slight promotion of collagen synthesis (Figure S8) observed in AgMOFN-CDs/illuminated cells could also be established. IL-1 was found to inhibit collagen biosynthesis by inhibiting the expression of insulin-like growth factor-I receptor (IGF-IR), the most potent stimulator of collagen biosynthesis in fibroblasts [46]. An observed decrease in IL-1 levels suggests a therapeutic pathway where AgMOFN-CDs combined with light could potentially mitigate its inhibition through the upregulation of IGF-IR expression and slight promotion of collagen biosynthesis in fibroblasts.

Since AgMOFN-CDs offer good NIR light harvesting capability, they could absorb the laser NIR light and transform it to heat, referred to as the photothermal effect, or emit light of a particular wavelength. Therefore, we wanted to test the emission and photothermal capacity of the hybrid nanomaterials after NIR illumination, which could be related to a mechanism that drives enhanced proliferation and wound closure. We recorded the emission of the solvent (physiological solution) as a control and solutions of AgMOFN-CDs and AgMOFS-CDs after 3 min of NIR laser illumination (16 mW/cm^2^). The emission of AgMOFN-CDs was two times higher than that of AgMOFS-CDs, which is in good correlation with the absorption characteristics of hybrid materials. The difference in emission is related to the difference in dopant atoms (nitrogen in N-CDs vs. sulfur in S-CDs). Nitrogen doping might create more favorable surface states or defect levels that facilitate NIR emission. Moreover, nitrogen doping introduces additional lone pair electrons, which could enhance the conjugation within the carbon lattice. This increased conjugation might facilitate a more efficient electron-hole pair separation and recombination, leading to improved NIR emission in AgMOFN-CDs. Consequently, AgMOFN-CDs absorb laser lights and emit additional NIR light that could further positively modulate the biochemical and molecular responses related to increased proliferation index and enhanced wound closure. On the other hand, the photothermal effect was excluded since a temperature rise was not detected during the 3 min illumination time with or without AgMOFN-CDs and AgMOFS-CDs treatments. The temperature was maintained at a range of 22.3 ± 0.1 °C throughout the entire irradiation period. Please note that we could not detect any photothermal effect for longer illumination time (6 min) and higher light intensity of 60 mW/cm^2^ (the maximal light intensity we could obtain from our Mantis laser after beam expansion). Light emission, which is higher in the case of AgMOFN-CDs, might contribute to the observed effect, but the emission does not lead to the photothermal effect.

The integration of FTIR spectroscopy into our investigation aims to enrich our understanding of the treatment’s impact on cellular proteins, according to their critical role in wound healing processes such as tissue formation, immune response, and cellular growth. Specifically, we analyzed the proteins to understand the structural modifications contributing to the observed cellular responses. SR-FTIR spectroscopy provided a detailed spectral signature that could reveal alterations in protein structures, such as changes in the Amide I and II bands, indicative of protein content and conformation, which are particularly evident with the synergistic application of NIR light and AgMOFN-CDs.

The increase in protein content observed in fibroblast cells stimulated by NIR light and/or AgMOFN-CDs treatment, Figure 8d, can be intricately linked to enhanced proliferation and migration. Proteins such as growth factors, cytokines, and those involved in the cell cycle and cytoskeletal reorganization are critical for these processes. As fibroblasts proliferate, they require a surge in cell cycle proteins to facilitate DNA replication and cell division. This increase in protein content, their localization in the central part of the cell, and the enlargement of cells observed in NIR-illuminated cells and cells subjected to combined treatment, Figure 8b,d, align with the cellular requirements for supporting larger cell volumes and the overall increase in biomass as cells prepare for proliferation. Similarly, migration necessitates the dynamic reorganization of cytoskeletal proteins, enabling the cell to move. Consequently, the elevated protein levels observed post-stimulation not only serve as markers of cellular activity but are directly implicated in the mechanisms driving fibroblast proliferation and migration, highlighting the role of protein synthesis in the cellular response to NIR light and/or AgMOFN-CDs treatment.

NIR light exposure also induces an increase in α-helices and β-turn, together with a decrease in cross-β structures, which is probably linked to alterations in cellular signaling pathways and metabolic activities that indicate a positive effect of photobiomodulation on enhanced cell viability [45,48].

The distinct separation of PCs over the PC2 axis between cells (Figure 6b) subjected to combined treatments versus those with individual treatments underscores the unique biochemical or structural changes induced by the synergistic or additive effects of AgMOFN-CDs and NIR light. Specifically, the increased prominence of α-helix structures in the combined treatment group (maximums PC2 at 1647 and 1542 cm^−1^ in loading plot Figure 6c) indicates that the combined treatment uniquely alters the protein landscape, beyond the alterations of individual therapies. This intermediate modification in protein structures indicates the unique cellular environment created by the combined treatment, where the effects of IR-induced photobiomodulation and the chemical-biological interaction of AgMOFN-CDs with cellular components converge. Such changes might enhance or modulate specific cellular functions, including proliferation, migration, and the synthesis of specific proteins in a manner distinct from each treatment’s effects alone. By stabilizing or promoting the formation of α-helices, such treatments may improve the function or expression of proteins that facilitate key steps in wound healing, including inflammatory response, new tissue formation, and remodeling. This implies a targeted enhancement of the wound healing machinery at the molecular level, potentially leading to more efficient and rapid tissue repair.

The absence of distinct clustering for AgMOFS-CDs treatments, coupled with the lack of clear differentiation between combined treatment (NIR illumination and AgMOFS-CDs) and NIR illumination alone, suggests that AgMOFS-CDs may not significantly alter the molecular features of cells in a distinguishable manner (Figure 6d,e), which was following the results of cell viability, proliferation, and migration. This difference in AgMOFN-CDs sets of experiments, where combined treatments and NIR illumination alone showed distinct effects, indicates a more pronounced or specific influence of N-CDs on cellular molecular characteristics.

The interaction of NPs with proteins is intricately influenced by their charge, size, and chemical composition. The introduction of positively charged N-CDs (zeta potential of +6.46) into AgMOFN-CDs, reducing their overall surface charge to −21, contrasts with the incorporation of negatively charged S-CDs, due to sulfonic/carboxylic groups into AgMOFS-CDs, resulting in a more negative charge of −54 [34]. This variation in charge characteristics diminishes electrostatic repulsion for AgMOFN-CDs and potentially alters their affinity for different protein domains, promoting more specific interactions with protein secondary structures, which is confirmed by disruption or unfolding of more organized structures, i.e., the destabilization of α-helices and promoting antiparallel β-sheet and random coil configurations (Figure 7). In addition, the more pronounced changes observed with AgMOFN-CDs treatment compared to AgMOFS-CDs could be attributed to the distinct ways nitrogen and sulfur interact with proteins. Nitrogen atoms from N-CDs might form hydrogen bonds with the carbonyl oxygen or amide hydrogen atoms in the protein backbone or electrostatic interactions with specific amino acid residues, potentially altering the protein structure more significantly than their sulfur-containing counterparts. In contrast, sulfur’s interactions, though relevant for disulfide bond formation in proteins, may not lead to as extensive conformational or functional changes when introduced through AgMOFS-CDs, resulting in less pronounced modifications.

## 5. Conclusions

In conclusion, our study showed the potential of NIR light-mediated AgMOFN-CDs’ wound healing potential alongside the antimicrobial effects previously reported. The results highlighted a synergistic impact with AgMOFN-CDs in combination with NIR light, leading to a remarkable increase in keratinocyte and fibroblast cell viability to nearly 150%, stimulation of the cell migration, viability, proliferation, protein, and collagen synthesis, in combination with the inhibition of production of pro-inflammatory cytokines. The most probable mechanism of the induction of the indicated changes is the specific structural and charge-driven interaction of nitrogen from N-CDs from AgMOFsN-CDs with proteins, which is augmented by the NIR light thanks to the optical properties of the nanocomposite system. This triple action (anti-inflammatory, antimicrobial, and targeted tissue repair), rooted in the principles of nanotechnology and targeted PDT, could be particularly advantageous in medical and cosmetic applications where accelerated healing and reduced inflammation are desired.

## Figures and Tables

**Table 1:** The concentration of IL-1 in indicated samples.

Sample	IL-1 Concentration (pg/mL)
Control	6.21
AgMOFS-CDs	8.84
AgMOFN-CDs	3.99
AgMOFS-CDs + illumination	5.862
AgMOFN-CDs + illumination	below the detection limit
Illuminated control	4.03

**Table 2:** Band assignments of MRC-5 spectra of the control and treated cell groups according to the Refs. [40,42].

Secondary Structure	Band Position (cm^−1^)
β sheet (antiparallel)	1692
β turns	1680 ± 3
α-Helix	1654 ± 2
Random coils	1642
β-Sheet (parallel)	1637 ± 2
cross-β	1627 ± 2

## Data Availability

Data are available upon reasonable request from the corresponding authors.

## Supplementary Materials

The following supporting information can be downloaded at: https://www.mdpi.com/article/10.3390/pharmaceutics16050671/s1, Figure S1: Viability assay of fibroblast cells in the absence of laser illumination (control group) and after 3 min laser illumination with fluences of 8, 16 and 60 mW/cm^2^. Figure S2: Viability assay of fibroblast cells in the absence of laser illumination (control group) and after laser illumination with fluences of 16 mW/cm^2^, laser exposure times were 3, 5, and 8 min. Figure S3: Viability assay of keratinocytes in the absence of laser illumination (control group) and after 3 min laser laser illumination with fluences of 60, 16, and 8 mW/cm^2^. Figure S4: Viability assay of human keratinocytes in the absence of laser illumination (control group) and after laser illumination with fluences of 16 mW/cm^2^, laser exposure times were 3, 5, and 8 min. Figure S5: The range of different concentrations of fibroblast (MRC5) treatments (AgMOFN-CDs NPs and AgMOFS-CDs NPs) from C1 to C5, 0.00125 mg/mL, 0.0006 mg/mL, 0.0003 mg/mL, 0.00015 mg/mL, and 0.000075 mg/mL. The graph shows the influence of different concentrations of treatments on fibroblast cell viability. Different shades of blue correspond to the AgMOFN-CDs NPs treatment, and shades of yellow correspond to the AgMOFS-CDs NPs treatment. **** *p* < 0.0001. Figure S6: The range of different concentrations of keratinocytes (HaCaT) treatments (AgMOFN-CDs NPs and AgMOFS-CDs NPs) from C1 to C5, 0.00125 mg/mL, 0.0006 mg/mL, 0.0003 mg/mL, 0.00015 mg/mL, and 0.000075 mg/mL. The graph shows the influence of different concentrations of treatments on keratinocyte cell viability. Different shades of blue correspond to the AgMOFN-CDs NPs treatment, and shades of yellow correspond to the AgMOFS-CDs NPs treatment. **** *p* < 0.0001. Figure S7: Effects of laser illumination and treatment with MOF–metal NPs on keratinocytes. The cells were illuminated with a laser fluence of 16 mW/cm^2^ within 3 min without and with AgMOFN-CDs NPs and AgMOFS-CDs NPs of concentration 0.0003 mg/mL. Figure S8: Collagen level estimated by hydroxyproline assay. Fibroblast cells were treated with AgMOFS-CDs and AgMOFN-CDs NPs and with a laser fluence of 16 mW/cm^2^ within 3 min. There are no statistically significant results.
