# Supplementary material for: Synergistic Enhancement of Targeted Wound Healing by Near-Infrared Photodynamic Therapy and Silver Metal–Organic Frameworks Combined with S- or N-Doped Carbon Dots
Source: Pharmaceutics. 2024 May 16;16(5):671. doi: 10.3390/pharmaceutics16050671 (PMC11124918; doi:10.3390/pharmaceutics16050671)
Supplement: Supplementary file 1 [file pharmaceutics-16-00671-s001.zip › pharmaceutics-2934966-supplementary.pdf]

Supplementary information

Synergistic Enhancement of Targeted Wound Healing by Near-Infrared Photodynamic Therapy and Silver Metal–Organic Frameworks Combined with S- or N-Doped Carbon Dots

Maja D. Nešić, Iva A. Popović, Jelena Žakula, Lela Korićanac, Jelena Filipović Tričković, Ana Valenta Šobot, Maria Victoria Jiménez, Manuel Algarra, Tanja Dučić and Milutin Stepić

S1. Setting up optimum experimental conditions

The first experiments were performed to find the most convenient and effective laser intensity and illumination time, which is short enough and positively influences cells' growth processes.

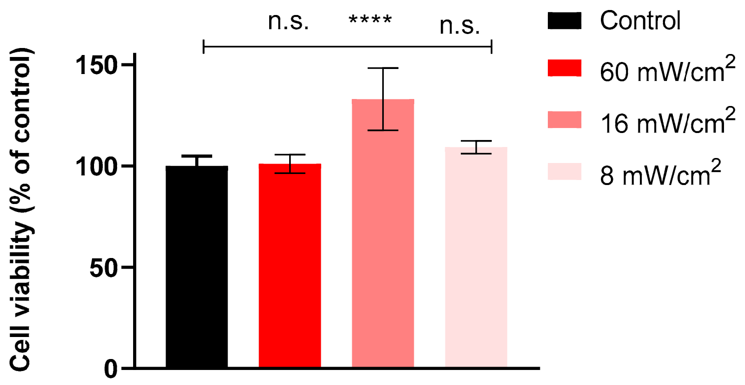

Figure S1. Viability assay of fibroblast cells in the absence of laser illumination (control group) and after 3 min laser illumination with fluences of 8, 16, and 60 mW/cm^2.

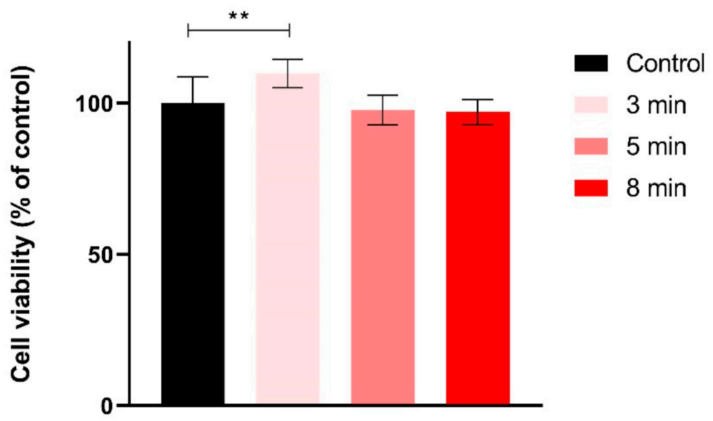

Figure S2. Viability assay of fibroblast cells in the absence of laser illumination (control group) and after laser illumination with fluences of 16 mW/cm^2, laser exposure times were 3, 5, and 8 min.

In the next set of experiments, the influence of laser beam intensity (Figures S1 and S3) and time of laser illumination (Figures S2 and S4) were tested on fibroblast and keratinocyte cells. Based on the results shown in Figures S1 and S3, a laser illumination with a fluence of 16 mW/cm^2 was used for further experiments with both cell lines because it gives higher or the same cell viability values compared to a lower or more intensive beam, respectively.

Furthermore, both cell lines exposed to laser illumination within 3 min had higher cell viability values than control (Figure S2 and S4). Based on the results in this set of experiments, it can be assumed that the 16 mW/cm^2 laser illumination within 3 minutes positively influenced both fibroblast and keratinocyte cell growth. Like the fibroblast cells, the cell viability of keratinocytes was also stimulated with the same parameters of laser illumination. Therefore, these parameters are used in further experiments.

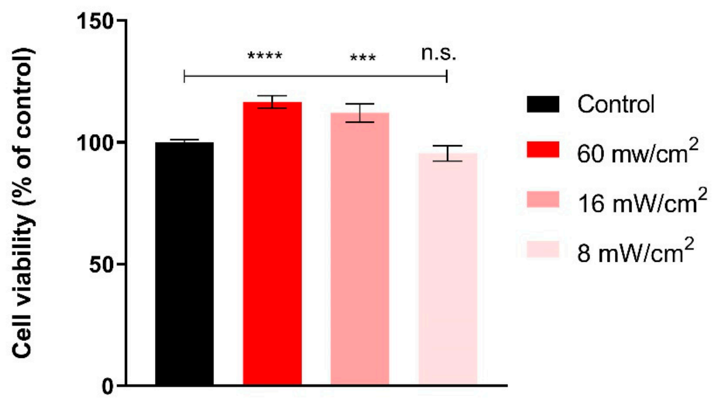

Figure S3. Viability assay of keratinocytes in the absence of laser illumination (control group) and after 3 min laser laser illumination with fluences of 60, 16, and 8 mW/cm^2.

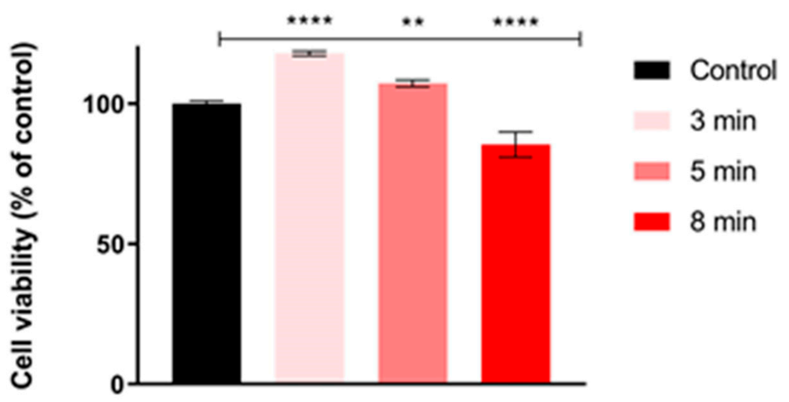

Figure S4. Viability assay of human keratinocytes in the absence of laser illumination (control group) and after laser illumination with fluences of 16 mW/cm^2, laser exposure times were 3, 5, and 8 min.

S2. The influence of different concentrations of treatments on cell viability

The cells, fibroblasts, and keratinocytes were treated with varying concentrations of AgMOFN-CDs NPs and AgMOFS-CDs NPs (0.00125 mg/mL, 0.0006 mg/mL, 0.0003 mg/mL, 0.00015 mg/mL, and 0.000075 mg/mL) to find the concentration to obtain the highest cell viability values. The results are presented in **Figures S5 and S6**. According to the results, for further experiments, the same concentration of 0.0003 mg/mL for both treatments (AgMOFN-CDs NPs and AgMOFS-CDs NPs) was chosen based on the results presented.

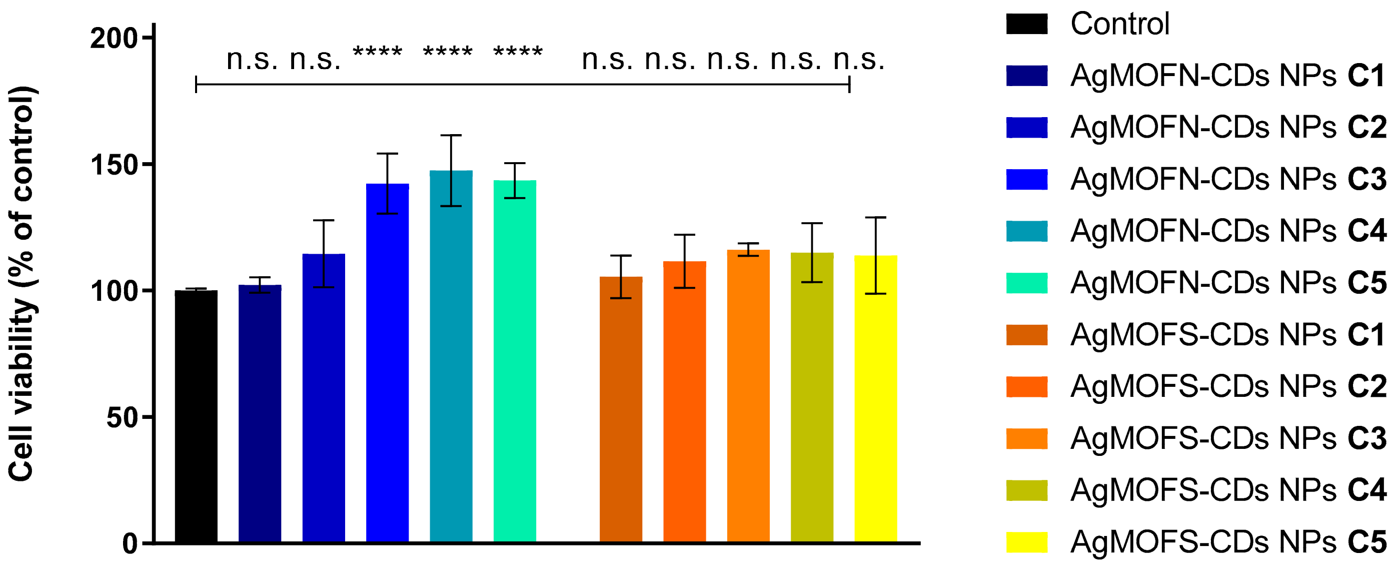

Figure S5: The range of different concentrations of fibroblast (MRC5) treatments (AgMOFN-CDs NPs and AgMOFS-CDs NPs) from C1 to C5, 0.00125 mg/mL, 0.0006 mg/mL, 0.0003 mg/mL, 0.00015 mg/mL, and 0.000075 mg/mL. The graph shows the influence of different concentrations of treatments on fibroblast cell viability. Different shades of blue correspond to the AgMOFN-CDs NPs treatment, and shades of yellow correspond to the AgMOFS-CDs NPs treatment. **** $p<0.0001$

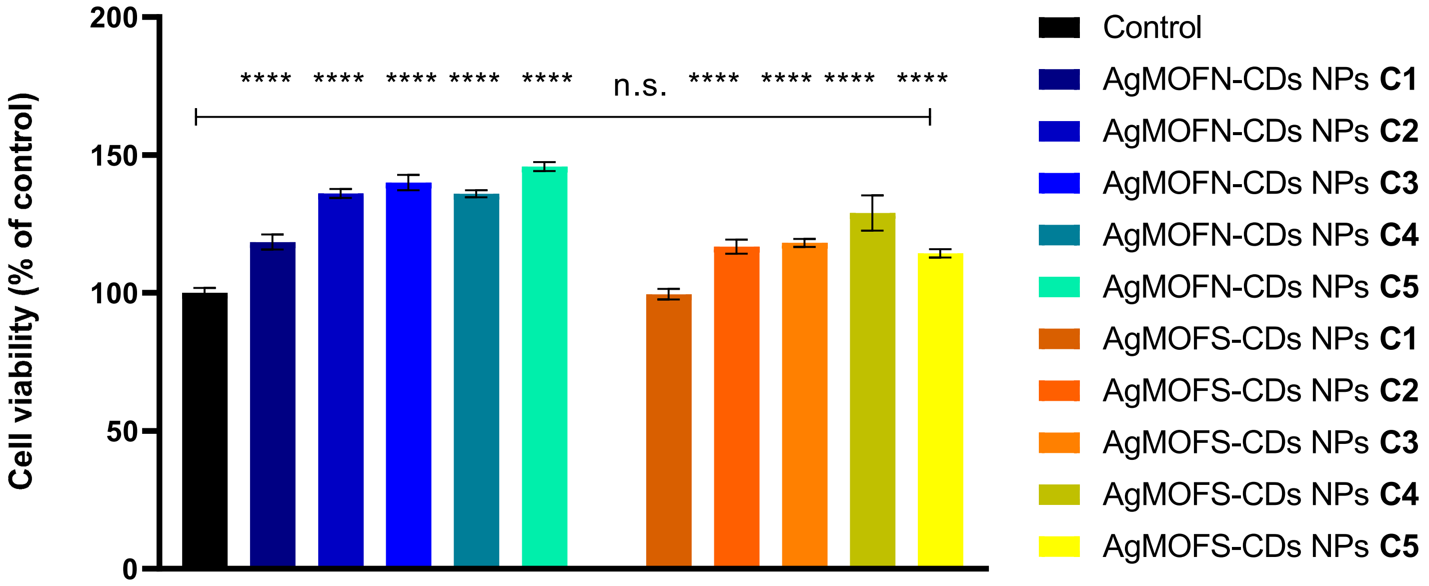

Figure S6: The range of different concentrations of keratinocytes (HaCaT) treatments (AgMOFN-CDs NPs and AgMOFS-CDs NPs) from C1 to C5, 0.00125 mg/mL, 0.0006 mg/mL, 0.0003 mg/mL, 0.00015 mg/mL, and 0.000075 mg/mL. The graph shows the influence of different concentrations of treatments on keratinocyte cell viability. Different shades of blue correspond to the AgMOFN-CDs NPs treatment, and shades of yellow correspond to the AgMOFS-CDs NPs treatment. **** $p<0.0001$

S3. Synergic NPs/light effect on cells

Figure S7 represent the results of the treatment and illumination of keratinocytes. The results show that combined treatment enhances cell viability compared to individual treatments for AgMOFN-CDs (AgN) and AgMOFN-CDs (AgS) and illumination. This enhancement in cell viability may be attributed to the combined effects of direct cellular stimulation by the nanoparticles and the photobiomodulatory impact of NIR light, which could promote cellular activities pivotal for keratinocyte function and survival.

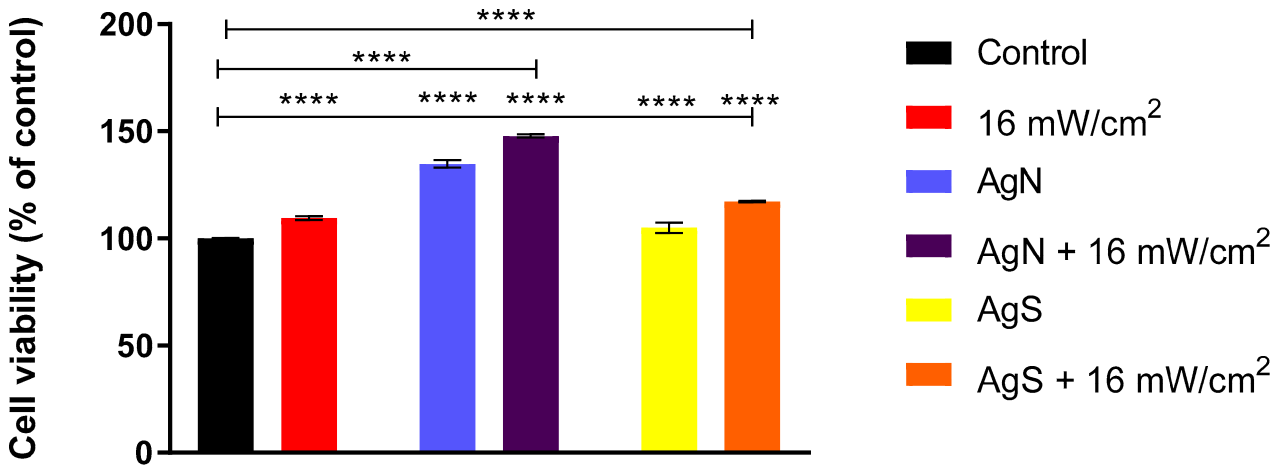

Figure S7. Effects of laser illumination and treatment with MOF–metal NPs on keratinocytes. The cells were illuminated with a laser fluence of 16 mW/cm^2 within 3 minutes without and with AgMOFN-CDs NPs and AgMOFS-CDs NPs of concentration 0.0003 mg/mL.

S4. Effect on the collagen production

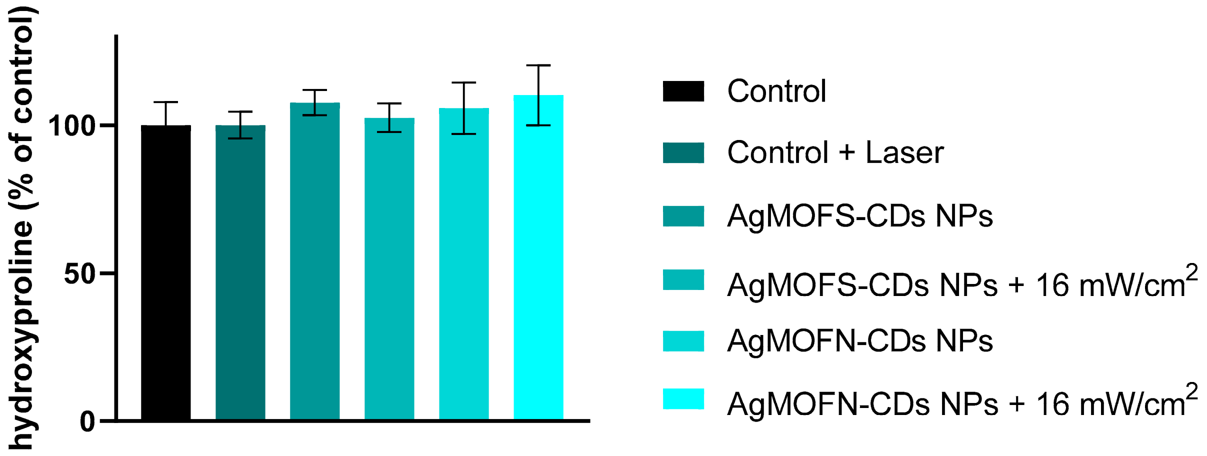

Figure S8. Collagen level estimated by hydroxyproline assay. Fibroblast cells were treated with AgMOFS-CDs and AgMOFN-CDs NPs and with a laser fluence of 16 mW/cm^2 within 3 min. There are no statistically significant results.
